# World carbon pricing database: sources and methods

**DOI:** 10.1038/s41597-022-01659-x

**Published:** 2022-09-17

**Authors:** Geoffroy Dolphin, Qinrui Xiahou

**Affiliations:** Resources for the Future, 1616 P St NW, Washington, DC 20036 USA

**Keywords:** Energy economics, Economics, Climate-change mitigation

## Abstract

This note describes the sources and methods used to construct the World Carbon Pricing Database (WCPD). This database contains a harmonized record of the sectoral coverage and prices associated with carbon pricing mechanisms implemented worldwide at the national and subnational levels over the period 1990–2020. The dataset follows IPCC 2006 sectoral disaggregation, which allows for a straightforward integration with other datasets following the same structure.

## Abstract (summary)

**Table:** 

Measurement(s)	Level of CO2 price
Technology Type(s)	Manual record

## Background & Summary

Over the last 30 years, the number of jurisdictions that have implemented a carbon pricing mechanism has grown significantly. Today, 43 national and 32 subnational jurisdictions have such a mechanism in at least one sector. However, a standardized and centralized record of the sectoral scope and prices applied to CO_2_ emissions by these mechanisms is lacking.

The initiative closest to what this dataset provides is the data collection effort led by the World Bank and made available through its Carbon Pricing Dashboard (*World Bank*^1^). However, it presents two shortcomings. First, the information available is structured around carbon pricing mechanisms (not jurisdictions). Despite a typical one-to-one mapping between a carbon pricing mechanism and a jurisdiction, this is not always the case (as with, for example, the EU ETS or the Regional Greenhouse Gas Initiative). Second, it is not disaggregated enough to allow for use in—and integration with—detailed analytical work.

This dataset provides an essential contribution to filling that gap. It covers mechanisms introduced since 1990 at the national and subnational levels and is the most comprehensive attempt at providing a systematic description of carbon pricing mechanisms in terms of their sectoral (and fuel) scope and the associated price signal. It should prove of interest to a wide range of parties, including academic researchers, policy analysts and interested civil society organizations.

A key feature of this dataset is that it provides information structured by territorial jurisdiction, not carbon pricing mechanism. This is achieved by mapping information available for each mechanism onto jurisdictions. This mapping accounts for the possibility that multiple mechanisms apply to the same emissions sectors and, in such instances, presents information separately for each mechanism (see details in section 3). It also covers a long period (1990–2020) and, hence, allows for (re)constructing time series of prices applied to emissions in the jurisdictions that had such prices. In addition, its disaggregation by IPCC 2006 sectors allows for a straightforward integration with other data sources following the same disaggregation (see section 5).

These features make the dataset a valuable tool to track the development of carbon pricing mechanisms. It also provides enough data to analyze their impact in a broad range of social, technological, and sectoral contexts. For instance, for the United States, it provides information on carbon pricing mechanisms in force in states arising from regional initiatives, such as the Regional Greenhouse Gas Initiative (RGGI), or state-level policies, such as the California cap-and-trade mechanism. Similarly, for national jurisdictions, it records the implementation of both regional initiatives, such as the EU emissions trading system (ETS), and national mechanisms targeting the same or other sectors.

This dataset has served as the backbone for two academic research projects. The initial publication associated with it is *Dolphin et al*.^2^. It is now made available as an open-source resource with the hope that it can be useful to many more, as well as benefit from feedback and contributions from a wider community. I also hope that this standardized reporting and assessment of carbon pricing mechanisms will provide easier access to information about them and improve transparency around their implementation.

The source code, written in Python 3, raw data files, and formatted dataset files and scripts are available at https://github.com/g-dolphin/WorldCarbonPricingDatabase under a CC0 1.0 Universal (CC0 1.0) Public Domain Dedication License (https://creativecommons.org/publicdomain/zero/1.0/). All files are also available under the same license on a dedicated Dryad repository. The digital object identifier associated with this repository is: 10.5061/dryad.547d7wmbq. A version of this manuscript describing an earlier version of the dataset is available at https://www.rff.org/publications/working-papers/world-carbon-pricing-database-sources-and-methods/.

The database records information on the institutional design and sectoral scope of mechanisms creating an explicit price on CO_2_ emissions at the sector-fuel level. It also records price(s) associated with each of these mechanisms. Information about both carbon taxes and emissions trading (cap-and-trade) mechanisms is recorded and presented separately. This dataset includes only pricing instruments whose design is directly related to the carbon content of fuels. Hence, it excludes energy taxes and duties and so differs from the structure of the data collected by the OECD to calculate the Effective Rates on Carbon (OECD, 2021a^3^).

The dataset currently covers 198 national and 94 subnational jurisdictions (50 US states, 13 Canadian provinces and territories, 31 Chinese provinces, autonomous regions and municipalities). Not all of these jurisdictions have a carbon pricing mechanism in force. The database could, however, accommodate information about pricing mechanisms in these jurisdictions, should one be implemented.

The data is disaggregated at the level of IPCC sectors following the IPCC 2006 guidelines for national greenhouse gas (GHG) emission inventories (*Buendia et al*.^4^; https://www.ipcc-nggip.iges.or.jp/public/2006gl/). For sectors in IPCC category 1 A (Fuel combustion activities), the dataset distinguishes between three aggregate fuel types: coal, oil, and natural gas.

The current dataset contains information on policy instruments targeting primarily CO_2_ emissions. In some instances, these instruments also cover other Kyoto greenhouse gases (and IPCC sectors). This coverage is accounted for in the present version. However, it does not incorporate information about carbon pricing mechanisms that primarily target non-CO_2_ Kyoto gases. For instance, Spain’s tax on Hydrofluorocarbons (HFCs) introduced in 2014 is not included. A future iteration of the dataset, currently in development, will integrate such information. A list of all the mechanisms included in the dataset, as well as the mechanisms’ identifiers, is provided in the file scheme_identifiers.csv in the following directory _raw/sources/. The list of mechanisms is reproduced in the Supplementary Information (SI2).

A related observation is that the price information provided (expressed per ton of CO_2_ equivalent) is best understood as applying to CO_2_ emissions within each IPCC sector. Usually, but not always, that same price also applies to emissions of non-CO_2_ Kyoto greenhouse gases.

The database is structured as follows. It has one *data* file and one *sources* file per jurisdiction, containing the actual data on carbon pricing mechanisms and a citation key that links directly to an entry in the bibliography (providing the details of the source from which the information was retrieved), respectively. The full list of references is available under the _dataset/sources/_references directory of the GitHub repository. Sources are grouped into five categories, so this directory contains five csv files (one for each category) with the bibliographic details (see section 4).

The data files are located at _dataset/data/ and are structured as follows. The first five columns are the “keys” of each database entry. The corresponding column titles are jurisdiction, year, ipcc_code, and Product. jurisdiction contains the jurisdiction’s name, year the year, ipcc_code the code of the IPCC emissions source category, and Product the name of the fuel product. Each row can be identified by a unique combination of values for these keys. The remaining columns in this file are the data records, described in the Supplementary Information (SI1).

As the table suggests, the dataset records information separately for carbon taxes and ETSs. Further, the database provides information about *all* pricing mechanism(s) applicable to a sector at the same point in time within a given jurisdiction; that is, the dataset includes as many tax- or ETS-relevant columns as there are applicable mechanisms, as some mechanisms are applicable to the same sector at the same time within a given jurisdiction. Across all jurisdictions with carbon pricing mechanisms, not more than two mechanisms have so far applied to the same sector at the same time within a given jurisdiction.

An objective of this dataset is to provide time series of coverage and prices applicable to emissions. Therefore, when, for a given row in the dataset, a new tax instrument (or ETS) is substituted for an existing one, the information (e.g., price) about this new mechanism will be recorded within the same column. The corresponding tax*_id or ets*_id value, however, will change.

For carbon taxes, the dataset also records separately sector-fuel specific price rebates: whether a given carbon tax regulation contains provisions for some sectors and/or fuels to be subject to a different tax rate. In practice, rebates implying a different price of CO_2_ across fuels are rare; more common are rebates set at the sector level and implying a different price of CO_2_ across sectors. See section 4.2.3 for more details.

Finally, an “NA” value in a *key* column of a given row means that the key is not applicable to that row, such as in the Product column: for all noncombustion IPCC  source categories, a distinction between fuel types is not applicable. The columns recording price level and rebates have an “NA” value if no pricing mechanism is in place (i.e., if the value of the corresponding coverage binary variable is set to 0).

## Methods

### Dataset compilation steps

The database is created by constructing a mapping of data on carbon pricing mechanisms to the national and subnational jurisdictions in which they are in force. It is constituted of two essential building blocks: (i) data on jurisdiction, sector, and fuel scope of each mechanism, and (ii) the tax rate and/or price of emission allowances at which these emissions are priced. Importantly, this data had not previously been systematically recorded using a standardized framework. As a result, dataset construction requires three steps (a visual representation of the dataflow is provided in the Supplementary Information, SI5):

1. ***Data collection***: information on each mechanism’s institutional design, sectoral scope and associated prices is collected from official government or secondary sources.
2. ***Data encoding***: the information collected is structured and encoded. Coverage information (jurisdiction, sector, fuel) is recorded in a Python script. Other institutional design features and price information are recorded in ad hoc csv files.
3. ***Dataset compilation***: the material created is used to generate the final dataset.

The compilation of the dataset happens in 5 steps, which are all contained in the db_build.py script:

1. Instantiate a dataframe containing the entire structure of the dataset; that is, the keys columns (jurisdiction, year, ipcc_cat_code, and Product), and all rows.
2. The coded scope information contained in ets_scope.py and taxes_scope.py, as well as the price information, extracted from the relevant raw csv files where they are recorded using the ets_prices.py and tax_rates.py scripts, are used to generate the following columns: tax, ets, tax*_id, tax*_rate_excl_ex_clcu, tax*_ex_rate, tax*_curr_code, ets*_id, ets*_price, ets*_curr_code.
3. Calculate tax*_rate_incl_ex_clcu by using files containing information on price rebates.
4. The dataset includes one additional step, calculating mechanism scope values for aggregate IPCC source categories based on the value for subsectors; these take the value 1 if and only if all subsectors are covered.
5. Finally, all variable entries for which the corresponding tax or ETS indicator value is 0 are set to “NA.”

All files (either csv or python scripts) that serve as input into the compilation of the final dataset and are mentioned above are described in the next section.

### Sources

The primary source of information on institutional design and coverage is legislative acts or related administrative acts from the competent jurisdictions. When such documents could not be retrieved (at this point), we relied on established secondary sources, such as official government publications or publications from international organizations, including the *State and Trends of Carbon Pricing* series published by the World Bank.

The price data is also primarily collected from legislative acts or related administrative acts. When such documents could not be retrieved or a more structured and harmonized data source was available, we relied on secondary sources. For emissions allowance prices, for instance, we used price series made available by the International Carbon Action Partnership (ICAP) through its Allowance Price Explorer (ICAP^5^). As indicated in section 4.2.2, prices are recorded in current local currency units (LCU). For some mechanisms, the legislation expresses the carbon price in a different currency (e.g., USD) than the currency of the jurisdiction to which it applies; if so, the price is recorded in that currency.

To retrieve legislative and administrative acts, we made extensive use of information available in the Climate Change Laws of the World database (www.climate-laws.org) and the OECD Database on Policy Instruments for the Environment (https://pinedatabase.oecd.org/) (OECD, 2021b^6^).

The dataset provides a reference to the source of each data point by including *sources* files for each jurisdiction that follow the same structure as the *data* files and include a citation key (and sometimes a comment) in each cell corresponding to a cell with information in the *data* files. All sources belong to one of the following source types: academic publication (journal), book (book), dataset (db), legislation (legislation), official government publication (gvt), report (report), or web page (web). The details of each source are recorded in the references csv file associated with the source category to which it belongs; respectively: _academic_papers.csv, _books.csv, _datasets.csv, _legislation.csv, _official_gov_publications.csv, _reports.csv, _webpages.csv.

Each source is assigned a citation key. The referencing structure combines the source type, citation key, and publication year, as follows: SourceType(CitationKey[Year]). For instance, the Sweden 1997 1A4C1 Coal entry of the tax variable contains the citation report(SMF-CT[2011]). The cell might also include a comment, separated from the reference by a semicolon. In our example, it is; underlying principle of the Swedish CO_2_ tax is that it applies to motor and heating fuels. All referenced documents have been accessed and made part of the dataset’s library.

## Data Records

This section describes the structure and encoding of the raw data. This data, together with the compiled dataset, is available at Dryad^7^.

### Scope

Scope dimensions: jurisdiction, year, IPCC source category, fuel, GHG.

All scope data is recorded in Python files located in the _raw/scope/ directory.

The information about each mechanism’s scope is encoded as a set of Python lists and dictionaries recorded in the taxes_scope*.py and ets_scope*.py files, respectively, for carbon taxes and ETSs. The * is a wildcard substituting for either _CO2, _CH4, _N2O or _F-GASES, the four (group of) gases covered in the dataset (extension of the dataset to non-CO_2_ gases is ongoing). Since the sectoral scope of a mechanism may vary by type of GHG it is easier to maintain separate records of sector-year scope of a mechanism for each GHG individually. Thus, the dataset records scope information separately for each GHG.

Each taxes_scope*.py or ets_scope*.py file offers lists containing the following: the jurisdictions to which the mechanism applies, IPCC source categories to which it applies, and fuels to which it applies. For each GHG, each scope dimension (jurisdiction, IPCC source category, fuel) has as many lists as there has been changes in the scope of the mechanism over its lifetime. These lists are respectively assigned to relevant years using dictionaries where “years” are dictionary keys, and the corresponding value is either the appropriate sector or fuel list. A description of the structure of the coverage encoding is presented in the Supplementary Information (SI4).

Exceptions to the general rules of each mechanism’s scope are recorded in taxes_scope_exceptions.py and ets_scope_exceptions.py, respectively for taxes and ETSs.

### Prices

The prices associated with each carbon pricing mechanisms are recorded in individual, mechanism-specific, csv files. The file naming rule is [scheme_identifier]_prices.csv. One price is recorded for each GHG separately. Usually (but not always), the same price applies across fuels and sectors for each GHG. The recorded price is the full price of emissions. Sector- or sector-fuel-level departures from this price (i.e., price rebates, see next section) are recorded in separate files.

For carbon tax mechanisms covering emissions from IPCC Energy sectors, the records distinguish between the rate applied to three aggregate fossil fuel categories (coal, oil, and natural gas). The rate, expressed in LCU/tCO_2_e, typically does not differ by category, with some exceptions (e.g., Norway). For ETSs (cap-and-trade), only one price value is recorded for each year, as the scope of the ETS is set at the sector level and associated emissions allowances cover emissions within covered sectors, regardless of the fuel from which they originate. The recorded value is either the yearly average of daily allowance prices or the allowance-weighted average of clearing prices in all auctions held within that year. Whether one or the other is recorded is primarily determined by the information publicly available, and which value is recorded is clearly indicated by a comment in the corresponding comment column of the files.

Finally, note that the carbon prices recorded in this database reflect the marginal, not average, price of emissions (OECD, 2021). Typically, if the price applies to the entire emission base, the average and marginal carbon prices do not differ. However, when tax-free emissions allowances or emissions permits are granted for free to sectors covered by a pricing mechanism, then a wedge between marginal and average price arises.

All price data are recorded in csv files located in the _raw/price/ directory.

### Price rebates

A rebate on the full price of emissions may be granted to particular sectors or fuels falling under the scope of a pricing mechanism. We denote these exemptions as price rebates because they grant a rebate on what is otherwise the full price on emissions but apply to all emissions within the scope of the mechanism. Such rebates occur in carbon taxes, not ETSs. In the latter case, all sectors and gases within the scope of the system face the same *marginal* price on their respective emissions. In an emissions trading system, industries may be granted some emissions allowances for free, such as in the early phases of most ETSs introduced so far. Such free allocation reduces the *average* price on emissions faced by covered emitters but not the marginal value of avoided emissions (OECD, 2021a).

Price rebates are currently manually recorded in separate csv files that follow the same structure (jurisdiction, year, sector, product) as the main data files. All price rebates data is recorded in csv files located in the _raw/price_rebates/ directory.

### Exemptions from scope

Exemptions from scope are regulatory provisions exempting some of a sector’s emitters (and associated emissions) from the scope of a particular instrument, and they include the following:

- *Compliance thresholds*, which exempt some emitters from the scope of a given mechanism based on their total yearly emissions, as for Chile’s carbon tax, or rated thermal input, as for the EU ETS; and
- *Administratively set exemptions if covered by another mechanism*, which may occur when the emissions might be covered by two mechanisms and the liability is waived for one of the two.

We record within-sector scope exemptions using a coverage factor. This factor is an initial attempt to account for administrative rules of implemented carbon pricing mechanisms affecting the scope of emissions covered within sectors. Coverage factors are recorded for each pricing mechanism in csv files located at _raw/coverage_factor/. Coverage factors are preliminary, and their development is ongoing.

The calculation (or direct encoding) of coverage factors is based on administrative data or information about the carbon pricing mechanisms. Our primary approach is to compare administrative emissions data (e.g., EU ETS registry data) to inventory data. In theory, the former is strictly smaller than the latter, as administrative data only includes emissions from covered entities. Two issues may arise, however. First, administrative data is not available for all mechanisms. For ETSs, such records exist as part of the emissions registries maintained. For carbon taxes, similar administrative records may exist showing which entity paid the carbon tax and for which amount of emissions. However, these are not publicly available.

Second, when it is available, methodological differences in the construction of administrative and inventory data create inconsistencies rendering a comparison difficult, at least at the sector level. Below, we describe how the coverage factors for various jurisdictions and sectors are set.

#### Emissions trading

In the current version of the dataset, coverage factors for ETSs are manually inputted based on secondary data sources about the mechanisms’ scope. In ongoing development, we attempt to calculate an adjustment based on the ratio of registry emissions to inventory emissions. As methodological differences in the construction of registry and inventory data currently preclude a consistent calculation at the sector level, we attempt to calculate this ratio at the jurisdiction level.

#### Taxes

The current default assumption is that taxes cover 100 percent of a sector’s emissions, because taxes apply to all entities consuming the fuel within that sector. Only in some specific cases is the coverage less than 100 percent:

- Singapore: compliance threshold, and
- Colombia: compliance threshold.

When a specific coverage factor could not be determined, 100 percent coverage was assumed.

### Overlapping mechanisms

Within a jurisdiction, two mechanisms typically do not apply to the same sectors; that is, they have no sectoral overlap in coverage. Mechanisms do sometimes overlap at the sectoral level, but this overlap does not extend to actual emissions within those sectors, as the mechanisms are designed to apply to different emissions within them. For instance, for countries participating in the EU ETS, their national carbon tax is designed to cover only emissions from installations that are not participating in the EU ETS.

However, an overlap sometimes exists. Overlap between carbon pricing mechanisms is accounted for by maintaining a csv file recording the bilateral overlap between mechanisms, at the sector level. This file (overlap_mechanisms.csv) is available at _raw/overlap/.

## Technical Validation

The data collection procedure involves several steps, including primary collection and quality assurance.

1. Standardized data collection process
  All sources upon which the dataset relies must be traceable. All data points in the dataset are directly linked to a data source through a reference citation structure. All reference details are recorded in dedicated reference lists.
  No source of information is *a priori* excluded from the set of admissible sources. However, sources that have a higher degree of reliability are preferred to sources with lower reliability. In the context of the present dataset, we consider primary legislation or, when applicable, secondary delegated administrative of executive acts, as the most reliable source of information about any given carbon pricing mechanism. Other sources are ranked in according to their degree of reliability of with regard to their *closeness* to that primary data source. The current ranking is as follows:
  Primary or secondary legislative acts; delegated acts > Reports or datasets from international organizations; official government publications > publications in academic journals; books; news; non-government reports; web; news articles
  The source documents identification process implemented gives priority to more reliable sources; that is, the search starts by looking for documents belonging to the first category, and only moves to the next category if no satisfactory source was identified.
2. Standardized document storage structure
  All downloaded documents are sorted and stored according to a standardized filing (directory) structure. The first node of that structure is the carbo pricing mechanism. Therefore, the structure is as follows:
  [scheme_identifier]/[document_type]
3. Data encoding framework and data processing flow
  The information contained in the identified documents is then manually encoded in either Python or csv files. Encoding in each file follows an established structure. The structure of these files is described in the Supplementary Information (SI4).
4. Quality assurance

The entire dataset generation pipeline is hosted on GitHub. This includes both the raw (manually encoded) data and the final dataset files, as well as the Python files implementing the transformation of the former into the latter. All modifications to the raw data files are executed on separate development branches of the repository and reviewed before integration into the main branch. The consistency of the change or update with the original data source is checked upon review.

The consistency of the output contained in the final dataset is checked against data contained in other datasets such as the World Bank Carbon Pricing Dashboard.

## Usage Notes

### Integration with other datasets

The structure of the dataset allows for a straightforward integration with other data sources that follow IPCC 2006 sectoral disaggregation or with data using different disaggregation, if used in combination with appropriate concordance tables. One such integration is with jurisdictions’ GHG emissions inventories, such as reported through the UNFCCC process and available through the UNFCCC data portal (https://di.unfccc.int/time_series) or estimated and compiled by institutions such as the Joint Research Centre of the European Commission and available in its Emissions Database for Global Atmospheric Research (EDGAR, https://edgar.jrc.ec.europa.eu/). For further details about EDGAR methodology, see https://edgar.jrc.ec.europa.eu/methodology.

In addition, for IPCC Energy source categories, the dataset provides a correspondence with the *Flow* (i.e., sector) key of the International Energy Agency’s CO_2_ emissions from fuel combustion data (*IEA*^8^). This facilitates integration with IEA data.

Combining this dataset with GHG emissions data provides several opportunities, including calculating the share of emissions covered by pricing mechanisms within national and subnational jurisdictions. It also allows for calculating emissions-weighted average carbon prices, at the sector or jurisdiction level. Such calculations were performed as part of a separate but related undertaking and resulted in emissions coverage figures and average prices at the jurisdiction level for 1990–2020. A companion paper describes the data sources and methodology related to these metrics.

### Planned updates and extensions

The dataset is under continuous development. Although every precaution has been taken to accurately record coverage and price information for each carbon pricing mechanism, the magnitude of the effort has been such that some inaccuracies might remain. Suggestions to update existing records and contributions to the extension of the dataset to other features of carbon pricing mechanisms are welcome; please refer to the guidelines available on the GitHub repository.

In addition, this dataset would benefit from the following extensions.

1. Update of data to the latest year to reflect institutional design and price changes pertaining to mechanisms, as well as information on mechanisms established since the last release.
2. Integration of information on the carbon pricing mechanisms in force in two Japanese municipalities: Saitama and Tokyo.
3. Extension to mechanisms covering other Kyoto GHGs than CO_2_ and recording of Kyoto gases covered by each mechanism.
4. Integration of information about tax-free allowances and free allocation of emission permits (by sector).

The next update of the dataset will focus on these extensions, with specific attention given to (3) and (4). Implementing the former will allow to provide a comprehensive summary of pricing mechanisms of all GHGs whereas implementing the latter will allow to calculate sector- or sector-fuel-level *average* – not *marginal* – carbon prices and account for tax-free emissions allowances (as in the case of, e.g., the South Africa carbon tax) and freely allocated emissions allowances in emissions trading systems in the calculation of the economy-wide average.

## Supplementary information

Supplementary Information

## Data Availability

All code is written in the Python 3 programming language. All files, including Python files, necessary to the compilation of the dataset are available on the following GitHub repository: https://github.com/g-dolphin/WorldCarbonPricingDatabase.
