# Supplementary material for: World carbon pricing database: sources and methods
Source: Sci Data. 2022 Sep 17;9:573. doi: 10.1038/s41597-022-01659-x (PMC9482624; doi:10.1038/s41597-022-01659-x)

Supplementary Information

Table of Contents

SI1 DATASET CONTENT **2**
SI2 PRICING MECHANISMS IDENTIFIERS **2**
SI3 CODING RULES **4**
SI4 DATABASE RAW FILES DESCRIPTION **5**
RAW DATA 5
AUXILIARY FILES 5
SI5 DATAFLOW REPRESENTATION **6**

SI1 Dataset content

Table 1 Description of variables

Variable name	Description	Values	Variable type
**tax**	A binary value indicating whether the sector(-fuel) is covered by at least one tax instrument.	{0,1}	integer
**ets**	A binary value indicating whether the sector(-fuel) is covered by at least one emissions trading system.	{0,1}	integer
**tax*_id**	Identifier of the applicable tax instrument.	-	string
**tax*_rate_excl_clcu**	Tax rate in current local currency unit (LCU) per ton of CO_2 equivalent.	[0,)	float
**tax*_ex_rate**	Rate of exemption applicable (e.g., 0.1 if a 10 percent reduction on the full rate applies); 0 if no exemption applies to that entry.	[0,1]	float
**tax*_rate_incl_ex_clcu**	Net tax rate (accounting for exemption) in current LCU per ton of CO_2 equivalent.	[0,)	float
**tax*_curr_code**	3-letter ISO code of the currency in which the tax rate is recorded (e.g., EUR for euro).	-	string
**ets*_id**	Identifier of the applicable emissions trading system	-	string
**ets*_price**	Price of an emissions allowance in current local currency unit per ton of CO_2 equivalent.	[0,)	float
**ets*_curr_code**	3-letter ISO code of the currency in which the allowance price is recorded (e.g., EUR for euro)	-	string

Note: The * in the variable names is a wildcard substituting for "" or "_2". As such, it allows to refer to identical columns for different implemented mechanisms at once.

SI2 Pricing mechanisms identifiers

Table 2 Carbon pricing mechanisms identifiers

**scheme_name**	**scheme_id**
Alberta TIER	can_ab_ets
Alberta carbon tax	can_ab_tax
Argentina carbon tax	arg_tax
Australia CPM	aus_tax
Baja California	mex_bc_tax
BC GGIRCA	can_bc_ggirca
BC carbon tax	can_bc_tax
Beijing pilot ETS	chn_bj_ets
California CaT	usa_ca_ets
Canada federal OBPS	can_obps
Canada federal fuel charge	can_tax_I

Canada federal fuel charge	can_tax_II
Chile carbon tax	chl_tax
China national ETS	chn_ets
Chongqing pilot ETS	chn_cq_ets
Colombia carbon tax	col_tax
Denmark carbon tax	dnk_tax
Denmark F-gases tax	dnk_tax_II
European Union ETS	eu_ets
Estonia carbon tax	est_tax
Finland carbon tax	fin_tax
France carbon tax	fra_tax
Fujian pilot ETS	chn_fj_ets
Germany ETS	deu_ets
Guangdong pilot ETS	chn_gd_ets
Hubei pilot ETS	chn_hb_ets
Iceland carbon tax	isl_tax
Iceland tax on F-gases	isl_tax_II
Ireland carbon tax	irl_tax
Japan carbon tax	jpn_tax
Kazakhstan ETS	kaz_ets
Korea ETS	kor_ets
Latvia carbon tax	lva_tax
Liechtenstein carbon tax	lie_tax
Luxembourg carbon tax	lux_tax
Massachusetts ETS	usa_ma_ets
Mexico carbon tax	mex_tax
Netherlands carbon tax - industry	nld_tax
Netherlands minimum CO2 price - electricity	nld_tax_II
New Brunswick carbon tax	can_nb_tax
New Brunswick OBPS	can_nb_ets
New Zealand ETS	nzl_ets
Newfoundland and Labrador PSS	can_nl_ets
Newfoundland and Labrador carbon tax	can_nl_tax
Northwest Territories carbon tax	can_nt_tax
Norway carbon tax	nor_tax_I
Norway carbon tax	nor_tax_II
Nova Scotia CaT	can_ns_ets
Poland carbon tax	pol_tax
Portugal carbon tax	prt_tax
Prince Edward Island carbon tax	can_pe_tax
Quebec CaT	can_qc_ets
Regional Greenhouse Gas Initiative	us_rggi
Saskatchewan OBPS	can_sk_ets

Shanghai pilot ETS	chn_sh_ets
Shenzhen pilot ETS	chn_sz_ets
Singapore carbon tax	sgp_tax
Slovenia carbon tax	slo_tax
South Africa carbon tax	zaf_tax
Spain carbon tax	esp_tax
Sweden carbon tax	swe_tax
Switzerland ETS	che_ets
Switzerland carbon tax	che_tax
Tamaulipas	mex_tm_tax
Tianjin pilot ETS	chn_tj_ets
UK carbon price floor	gbr_tax
UK ETS	gbr_ets
Ukraine carbon tax	ukr_tax
Virginia ETS	usa_va_ets
Zacatecas carbon tax	mex_za_tax

SI3 Coding rules

- **Price differences within fuel type aggregates:** Different tax rates may apply to different fuels within the main fuel categories (i.e., coal/peat, natural gas, oil). This comes in the form of differentiated applicable tax rates or varying exemption rates, such as for Mexico. In such cases, the value in the dataset is the highest rate applicable to the fuel category. The fuel- and/or sector-fuel-specific rates are recorded in the *scheme notes* for each scheme.
- **Encoding of partial and full sectoral exemptions:** If a sector is fully exempted from a given pricing mechanism, the coverage variable value is set to 0 for the sector (and all fuel categories, when relevant). If only a specific fuel type within a sector is exempted (partially or totally), then the coverage variable value is set to 1 for the sector (and all fuel categories, when relevant) and the tax_exemption_rate variable value is set to the value corresponding to the level of the exemption (which is 1 if exemption is total).
- **Price rebates exemptions**: If the exemption varies within the sector-fuel level, the highest exemption rate is recorded. For example, farmers in Alberta can receive up to 100 percent carbon levy rebates, depending on their eligibility. In such cases, the coverage variable in the corresponding row of the dataset and the exemption rate are set to 1.
- **Currency of price record in multi-jurisdiction mechanisms:** When a jurisdiction (or some sectors therein) is part of a multi-jurisdiction mechanisms, the price is expressed in the common currency of the mechanism and the recorded currency code is consistent with that currency. For instance, for the California-Quebec(-Ontario) cap-and-trade system, the price of allowances is expressed in US dollars (USD).
- **Recording prices in incomplete years**: if a mechanism was not in place for the entirety of a given year, a price (tax rate or allowance price) "pro-rata tempore" is calculated; the recorded price is a weighted average of all prices to which emissions were subjected throughout the year. This applies to all years apart from years in which a mechanism was first introduced. In that case, the recorded price and coverage are recorded "as if" it had been applied from January 1 of that year.
- **Multiple mechanisms**: when two mechanisms apply to the same sector, information on both mechanisms is recorded through additional columns in the dataset.
- **Coverage records of IPCC sectors that are exclusively sources of non-CO_2 GHG emissions**: if a policy instrument that targets CO_2 emissions also covers other Kyoto GHG,

then all IPCC sectors covered by that instrument are recorded. However, information about instruments that exclusively target non-CO_2 GHG emissions has not yet been recorded.
- **Partial price rebates on certain emissions within sectors:** In some cases (e.g., the Ireland carbon tax), the tax does not cover all emissions of a sector and a rebate is granted on emissions covered by the tax. If so, a combination of (i) the share of emissions it applies to (`cf_co2`) and (ii) the exemption rate allows to accurately record the instance.

SI4 Database raw files description

Raw data

Scope: coverage information in encoded in the `ets_scope*.py` and `taxes_scope*.py` files. The structure of these records is described below:

```
--------------------------------scheme_name------------------------------

# List(s) of jurisdictions

scheme_id_jur_* = []

# List(s) of IPCC sectors covered

scheme_id_ipcc_* = []

# List(s) of fuel categories

scheme_id_fuel_* = []


# Scope dictionaries

scheme_id_jur_cov = {year1: scheme_id_jur, …,2021: scheme_id_jur}

scheme_id_ipcc_cov = {year1: scheme_id_ipcc, …,2021: scheme_id_ipcc}

scheme_id_fuel_cov = {year1: scheme_id_fuel, …,2021: scheme_id_fuel}

-------------------------------------------------------------------------
```

Prices: When price data is manually encoded, it is saved in individual (i.e., one per pricing mechanism) `csv` files. The file naming convention is `[scheme_id]_prices.csv`. Each file follows the same structure:

- *Carbon taxes*: `scheme_id, year, ghg, product, rate, currency_code, source, comment`
- *ETSs*: `scheme_id, year, ghg, allowance_price, currency_code, source, comment`

When price data is retrieved from structured datasets, it is extracted directly from there and reformatted in the Python script.

Price (tax) exemptions: Exemptions are recorded manually *by jurisdiction* (not by pricing mechanism, as exemptions are, for the most part, jurisdiction rather than mechanism specific) in individual `csv` files. The file naming convention is [tax_ex_jurisdictionName].

Auxiliary files

- `IPCC2006-IEA-category-codes.csv` provides a mapping between IPCC sector names, their associated code and the corresponding International Energy Agency sector code. This

latter file is particularly useful to update the dataset, as its .csv files only include IPCC sector codes.

- `scheme_identifiers.csv` provides a correspondence between the names of pricing mechanisms' and their internal database identifier.

SI5 Dataflow representation

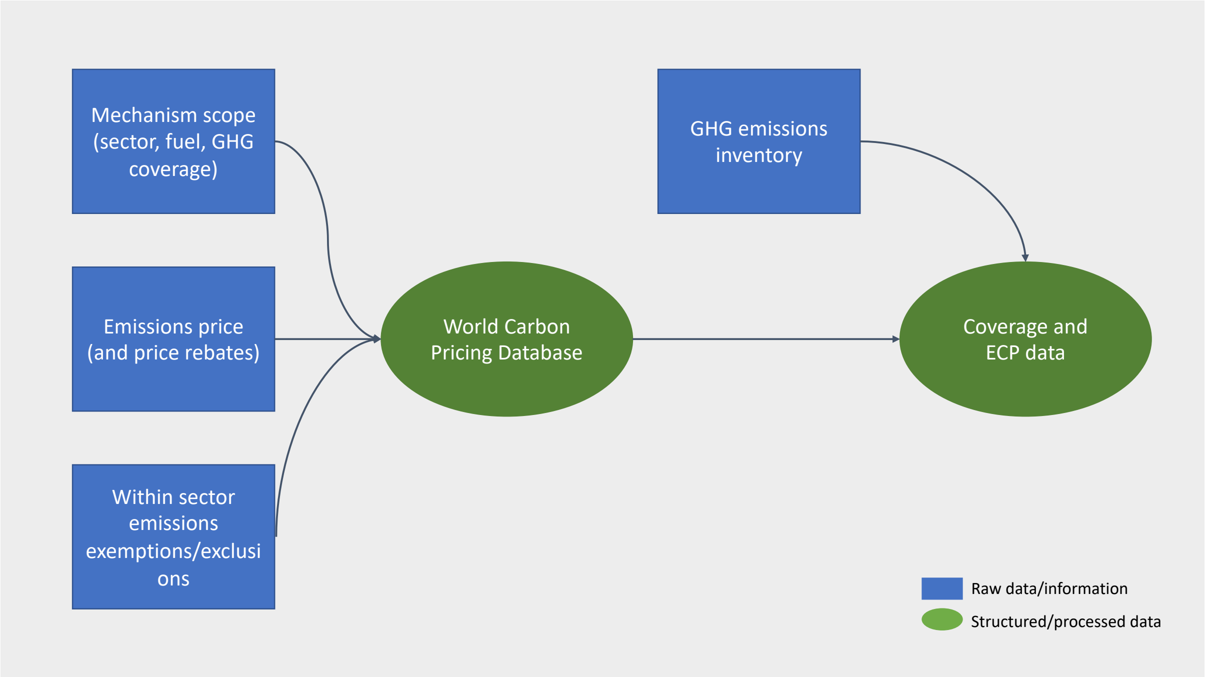

Supplement: Supplementary file 1 — Supplementary Information [file 41597_2022_1659_MOESM1_ESM.pdf]
